# Twenty-eight-week results from the REALISTIC phase IIIb randomized trial: efficacy, safety and predictability of response to certolizumab pegol in a diverse rheumatoid arthritis population

**DOI:** 10.1186/s13075-015-0841-9

**Published:** 2015-11-15

**Authors:** Michael E. Weinblatt, Roy Fleischmann, Ronald F. van Vollenhoven, Paul Emery, Tom WJ Huizinga, Maurizio Cutolo, Désirée van der Heijde, Benjamin Duncan, Owen Davies, Kristel Luijtens, Maxime Dougados

**Affiliations:** Brigham and Women’s Hospital, 75 Francis Street, Boston, MA 02115 USA; Department of Internal Medicine, University of Texas Southwestern Medical Center, 8144 Walnut Hill Lane, Dallas, TX 75231 USA; Karolinska Institute, Solnavägen 1, 171 77 Stockholm, Sweden; Leeds Institute of Rheumatic and Musculoskeletal Medicine, University of Leeds, Chapel Allerton Hospital, Chapeltown Road, Leeds, LS7 4SA UK; NIHR Leeds Musculoskeletal and Biomedical Research Unit, Leeds Teaching Hospitals NHS Trust, Great George Street, Leeds, LS1 3EX UK; Department of Rheumatology, Leiden University Medical Centre, Albinusdreef 2, 2333 ZA Leiden, The Netherlands; Research Laboratory and Academic Unit of Clinical Rheumatology, University of Genova, Viale Benedetto XV, 6, 16132 Genova, Italy; UCB Pharma, 8010 Arco Corporate Drive, Raleigh, NC 27617 USA; UCB Pharma, Allée de la Recherche 60, 1070 Brussels, Belgium; Paris-Descartes University, 12 Rue de l’École de Médecine, 75006 Paris, France; Cochin Hospital, 27 Rue du Faubourg Saint-Jacques, 75014 Paris, France

**Keywords:** Rheumatoid arthritis, Anti-TNF, DMARDs (biologic)

## Abstract

**Introduction:**

This 28-week, phase IIIb study assessed safety and maintenance of response to certolizumab pegol (CZP) in a diverse population of rheumatoid arthritis (RA) patients, stratified by prior anti-TNF exposure, concomitant methotrexate (MTX) use and disease duration. The ability to predict achievement of low disease activity (LDA) at week 28 from improvements in Disease Activity Score 28 (DAS28), erythrocyte sedimentation rate (ESR), swollen joint count (SJC) and Clinical Disease Activity Index (CDAI) up to week 12 was assessed.

**Methods:**

The 28-week study population included all patients who completed the double-blind (DB) phase and entered the open-label (OL) phase, receiving 200 mg CZP every 2 weeks (Q2W) ≥16 weeks. In the 12-week DB period, patients with active RA and an inadequate response to ≥1 disease-modifying antirheumatic drug (DMARD) were randomized 4:1 to CZP (400 mg at weeks 0, 2 and 4 then 200 mg Q2W) or placebo (Q2W), stratified by prior anti-TNF use, concomitant use of MTX and disease duration (<2 years vs. ≥2 years).

**Results:**

A total of 955 patients entered the OL phase. At week 28, similar clinical improvements were seen in those receiving CZP throughout (CZP → CZP; n = 771) and those receiving placebo during the DB phase and switching to CZP in the OL phase (placebo → CZP; n = 184) (ACR20 response rate = 59.7 % vs. 53.3 %; ACR50/ACR70 response rates were also similar). Effect of CZP treatment was similar regardless of prior anti-TNF use, disease duration and concomitant DMARDs, based on ACR20 response rates. The percentage of patients achieving DAS28(ESR) LDA at week 28 was calculated for DAS28(ESR), SJC or CDAI responders at earlier time points. Reductions from baseline (Δ) of DAS28(ESR) <1.2, ΔSJC <25 % or ΔCDAI <10 by week 12 were associated with <9 % chance of achieving LDA at week 28 regardless of prior anti-TNF exposure. Adverse event rates were similar for placebo → CZP and CZP → CZP patients, with no new safety signals identified.

**Conclusions:**

A diverse population of RA patients with varying disease duration showed rapid and sustained clinical improvements on CZP treatment, regardless of prior anti-TNF or concomitant DMARD use. Failure to achieve improvements in DAS28(ESR), SJC or CDAI within the first 12 weeks of CZP therapy was associated with a low chance of achieving LDA at week 28. No new safety signals were observed.

**Trial registration:**

ClinicalTrials.gov, NCT00717236, 15 July 2008

**Electronic supplementary material:**

The online version of this article (doi:10.1186/s13075-015-0841-9) contains supplementary material, which is available to authorized users.

## Introduction

Study populations in conventional clinical trials for anti-tumor necrosis factor (TNF) agents in rheumatoid arthritis (RA) have comprised a largely homogeneous patient group with high disease activity and no prior anti-TNF experience, reflecting a small proportion of patients in routine clinical care [1–5]. While such trials demonstrate the ability of anti-TNFs to slow radiographic progression and improve the signs and symptoms of RA in patients with active disease, studies which permit the inclusion of more diverse patient groups such as those with and without prior anti-TNF use, with and without baseline methotrexate (MTX) use or with prior exposure to non-MTX conventional synthetic disease-modifying antirheumatic drugs (csDMARDs) would more accurately reflect patients in real-world clinical care. The REALISTIC trial is one such study, from which the efficacy of certolizumab pegol (CZP) after 12 weeks of treatment in a heterogeneous population of RA patients has previously been reported [6]. Following the 12-week double-blind (DB) period, the REALISTIC study included a 16-week open-label extension (OLE) period, in order to assess the efficacy and safety of CZP as an add-on therapy to current treatment in this varied population relevant to clinical care, over a 28-week period.

Here we report the safety and key secondary outcomes, including maintenance of response to certolizumab pegol (CZP) in this heterogeneous population of RA patients, stratified by prior exposure to anti-TNFs, concomitant MTX use and disease duration. In addition, the results of exploratory analyses are reported, investigating whether lack of response during the first 12 weeks of treatment (based on the timing and magnitude of the change in Disease Activity Score based on 28 joints and erythrocyte sedimentation rate [DAS28(ESR)], swollen joint count [SJC] or Clinical Disease Activity Index [CDAI]), predicts failure to achieve low disease activity (LDA) at week 28 in this diverse patient population.

Early prediction of clinical outcomes allows identification of a time at which it might be appropriate to stop therapy in patients failing to respond to CZP and could allow an earlier change to a more appropriate treatment option, resulting in faster improvements in patient outcomes and potentially better cost efficiency for the health care system. It has been previously reported in the RAPID 1 trial for CZP that a lack of improvement in DAS28(ESR) by week 12 in RA patients predicted failure to achieve LDA at later time points [7].

## Methods

Full inclusion and exclusion criteria were reported in the primary publication [6]. Briefly, eligible patients were ≥18 years of age, had adult-onset RA for ≥3 months as defined by the 1987 American College of Rheumatology (ACR) criteria, and showed an unsatisfactory response or intolerance to ≥1 csDMARD [8]. Patients had active disease by ≥5 tender and ≥4 swollen joints (TJC; SJC [28-joint count]) and either C-reactive protein (CRP) ≥10 mg/l or ESR (Westergren method) ≥28 mm/h at screening. Biologic disease-modifying antirheumatic drugs (bDMARDS) were discontinued before study entry; patients were excluded if previously treated with >2 anti-TNFs, rituximab or abatacept. Analgesics, nonsteroidal anti-inflammatory drugs (NSAIDs), oral corticosteroids (10 mg/day prednisone equivalent) and csDMARDs, MTX, leflunomide, sulfasalazine, chloroquine, hydroxychloroquine, azathioprine and gold were permitted if doses were stable prior to baseline. Exclusion criteria included any other inflammatory arthritis or a history of chronic, serious or current infection (including evidence of latent tuberculosis [TB] defined as a positive purified protein derivative skin test [≥5 mm] or close contact with individuals with active TB). Patients positive for purified protein derivative could be included if active TB was ruled out and if they were adequately treated for latent TB (e.g., isonicotinic acid hydrazide), with treatment initiated at least 1 month before first administration with the study drug.

### Study design

This 28-week, phase IIIb study (NCT00717236) consisted of a 12-week, randomized, DB period and a subsequent 16-week OL phase. Patients were recruited from 230 centers in the USA and Canada (75 %), and Europe (25 %). The study protocol was approved by the Institutional Review Board/Independent Ethics Committee as defined in local regulations and performed according to the Declaration of Helsinki (please see Acknowledgements for a complete list of ethics committees that approved this study). All patients provided written consent. Sample size was selected to provide at least 90 % power to show a statistically significant difference between the active and placebo groups at week 12 in both the primary outcome (ACR20 response rate) and also subgroup analyses (prior anti-TNF usage; concomitant MTX treatment and disease duration).

**Fig. 1:**
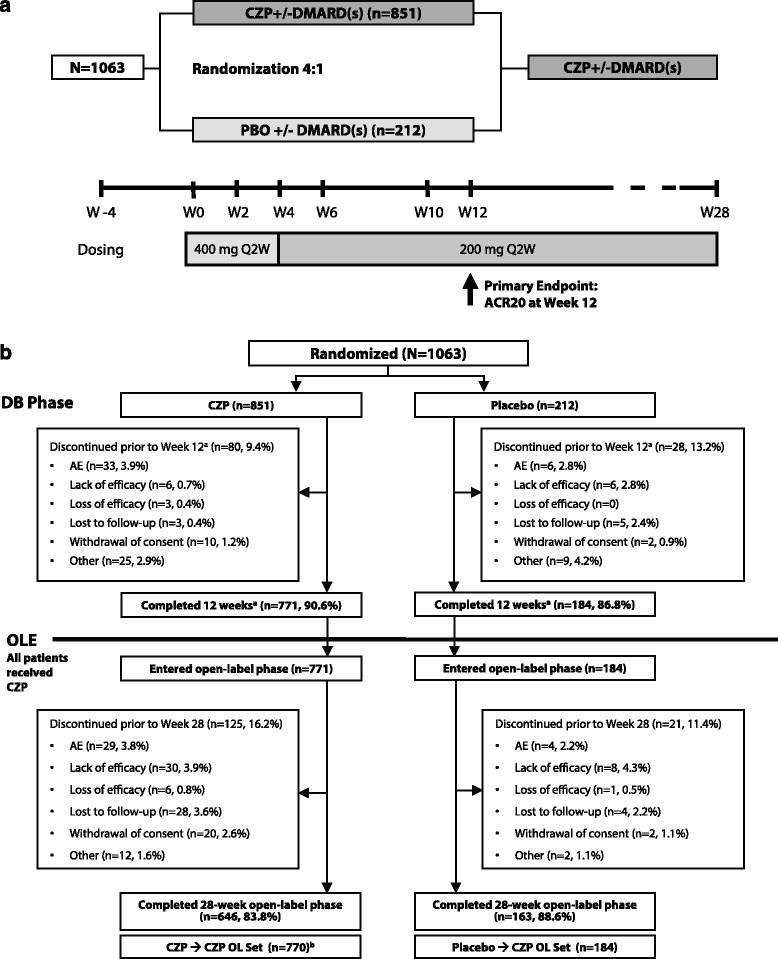
**a** REALISTIC trial design. **b** Subject disposition. ^a^DB phase. ^b^One week 12 CZP completer discontinued after week 12 due to an AE, did not receive any study medication in the OL phase, and was not included in the OL analysis set. *AE* adverse event, *CZP* certolizumab pegol, *DMARD* disease-modifying antirheumatic drug, *OLE* open-label extension, *Q2W* every other week

During the initial 12-week period patients were randomized 4:1 by means of an interactive voice response system to either 400 mg CZP at weeks 0, 2 and 4 followed by 200 mg CZP every 2 weeks (Q2W) (CZP patient group), or placebo (0.9 % saline) Q2W (placebo group), stratified by prior anti-TNF use, concomitant use of MTX and disease duration (<2 years vs. ≥2 years) to ensure balanced treatment assignments [6]. Patients who had completed 12 weeks of treatment with either 200 mg CZP Q2W or placebo were eligible to receive OL CZP 200 mg Q2W for ≥16 weeks (Fig. 1a). Results from the 16-week OL phase are reported here, together with the association between DAS28(ESR) low disease activity at week 28 and failure to achieve early responses.

### Efficacy and safety evaluations

Efficacy and safety evaluations were performed every 8 weeks until patient completion or withdrawal from the study. The primary endpoint of the study was ACR20 response rate at week 12 [6]. Secondary endpoints included efficacy measurements (ACR20/ACR50/ACR70 response rates, DAS28[CRP], Health Assessment Questionnaire-Disability Index [HAQ-DI], Clinical Disease Activity Index [CDAI] and Simplified Disease Activity Index [SDAI]) at week 12 and throughout the OLE.

Post hoc analyses included week 28 ACR20, ACR50 and ACR70 response rates stratified by prior anti-TNF use, and week 28 ACR20 response rates stratified by number and type of concomitant DMARDs at baseline, baseline MTX use, disease duration and rheumatoid factor (RF) titer at baseline. Post hoc analyses to predict the proportion of CZP-treated patients who achieved DAS28(ESR) LDA (≤3.2) at week 28 based on early responses were also conducted, stratified by prior anti-TNF experience. Failure to achieve LDA was predicted, based on the timing and magnitude of nonresponse in patients who did not achieve a reduction of <0.6, <1.2 and <1.8 units from baseline in DAS28(ESR) or SJC percentage reduction of <10 %, <25 % and <50 % from baseline or reduction of <10 CDAI from baseline at any time ‘up to’ and ‘at’ weeks 2, 6, or 12.

Adverse events (AEs) were recorded at each visit. Any events meeting the regulatory definition of a serious AE (SAE) [9], all opportunistic infections, malignancies (excluding some basal cell carcinomas at the discretion of the investigator) and any medical event assessed as being relevant by the investigator, including events that did not require hospitalization, were considered SAEs.

### Statistical analysis

Efficacy analyses up to week 28, and safety evaluations from week 12 up to 28, were performed on the OL set, comprising all patients who completed 12 weeks of treatment in the DB phase and who received ≥1 dose of OL CZP. ACR response rates were determined using nonresponder imputation (NRI) when patients withdrew for AE or lack or loss of efficacy, and last observation carried forward (LOCF) in case of any other reason. Least squares means (change from baseline) in DAS28(CRP), SJC and HAQ-DI were analyzed using a mixed-effects model for repeated measures (MMRM) to estimate response, which included terms for visit, visit by treatment interaction and baseline response (for the respective endpoint) by visit interaction. Predictability analyses by early changes in DAS28(ESR), SJC and CDAI were conducted using observed data (from the OL set); data for DAS28(ESR) were also analyzed using LOCF-imputed data from the full analysis set (FAS) (comprising all patients who received ≥1 dose of CZP). If all weeks prior to the specified time point were missing, the patient was not counted. Failure to achieve the specified reductions in DAS28(ESR), SJC and CDAI was defined as (percentage) change from baseline less than the cut point at every visit up to and including the time point under analysis. Predictability analyses by DAS28(ESR) nonresponse ‘at’ (as opposed to ‘up to’) the relevant time point were also conducted using LOCF-imputed data from the FAS. Out of the above defined populations, the number (percentage) of patients achieving LDA at week 28 is presented.

## Results

### Patients

A total of 1,063 patients were randomized into the DB phase of the study; 851 and 212 patients in the CZP and placebo groups, respectively. A total of 955 patients completed this 12-week phase and entered the OL phase; 771 (90.6 %) of the CZP group and 184 (86.8 %) of the placebo group (Fig. 1b). Of these, 809 (84.7 %) remained in the study until week 28. The median number of doses received during the OL phase alone, not including prior exposure during the DB phase, was 8 (range 1–21) for placebo → CZP patients and 8 (range 1–32) for CZP → CZP patients.

Patient demographics, baseline disease characteristics and prior and concomitant therapies were similar between placebo and CZP patients randomized in the DB phase [6] (and the placebo → CZP and CZP → CZP groups at entry to the OL phase (Table 1). Additionally, characteristics including age, disease duration and disease activity (measured by HAQ-DI, DAS28[ESR] and DAS28[CRP]) were similar when patients were stratified by prior anti-TNF use, concomitant MTX use at baseline and disease duration. The overall proportion of patients with prior anti-TNF use was similar in the patients present at entry into the DB phase and at the start of OL phase of the study (37.6 % vs. 37.4 %, respectively).

**Table 1:** Demographics and disease characteristics at baseline of the double-blind phase (OL set)

	OL set	
	Placebo → CZP^a^	CZP → CZP^a^
	(n = 184)	(n = 770)
Age (years), mean (SD)	53.7 (12.7)	55.3 (12.3)
Gender, % female	79.3	77.9
Disease duration (years), mean (SD)^b^	8.9 (9.0)	8.5 (8.7)
Disease duration <2 years, n (%)	45 (24.5)	184 (23.9)
Tender joint count, mean (SD)	14.7 (6.4)(n=184)	14.7 (6.5)(n=769)
Swollen joint count, mean (SD)	11.1 (5.1)(n=184)	11.8 (5.5)(n=769)
DAS28(CRP), mean (SD)	5.7 (0.8)(n=183)	5.7 (0.9)(n=768)
DAS28(ESR)	6.4 (0.8)(n=183)	6.4 (0.9)(n=767)
<3.2, n (%)	0	0
3.2–5.1, n (%)	11 (6.0)	68 (8.9)
>5.1, n (%)	172 (94.0)	699 (91.1)
HAQ-DI, mean (SD)	1.6 (0.6)	1.5 (0.6)
CRP (mg/L), median (min, max)	10.0 (2.9, 159.0)	9.0 (1.0, 164.0)
Rheumatoid factor positive (≥14 IU/mL), n (%)	127 (77.0)(n=165)	505 (73.9)(n=683)
Treatment history, n (%)		
Prior DMARD use	115 (62.5)	514 (66.8)
Prior anti-TNF	71 (38.6)	286 (37.1)
Prior anti-TNF/other biological use	76 (41.3)	309 (40.1)
Other prior medications^c^	51 (27.7)	209 (27.1)
Concomitant^d^ medications		
DMARDs	152 (82.6)	649 (84.3)
MTX	128 (69.6)	533 (69.2)
Other medications^c^	184 (100)	768 (99.7)

*OL* open-label, *CZP* certolizumab pegol, *DAS28* Disease Activity Score in 28 joints, *CRP* C-reactive protein, *ESR* erythrocyte sedimentation rate, *HAQ-DI* Health Assessment Questionnaire-Disability Index, *DMARD* disease-modifying antirheumatic drug, *TNF* tumor necrosis factor, *MTX* methotrexate

^a^Patients who completed 12 weeks of treatment with either CZP 200 mg every other week (Q2W) or placebo during the double-blind phase entered the OL phase and subsequently received active treatment (CZP 200 mg Q2W)

^b^Duration at screening visit

^c^Medications other than DMARDs, anti-TNFs or other biologics, including those for treatment of comorbidities

^d^Concomitant medications include those ongoing at the week 12 visit or taken during the OLE phase

### Treatment efficacy

**Fig. 2:**
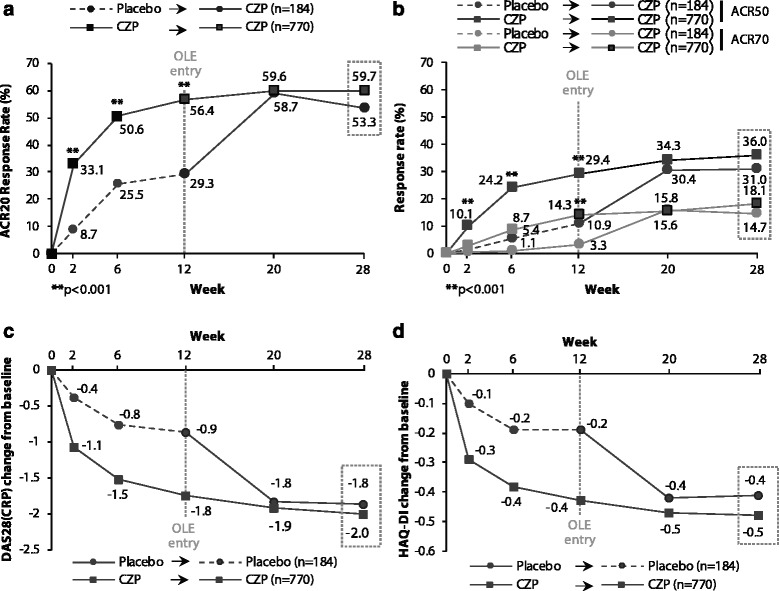
Treatment response to week 28. **a** ACR20 response rates up to week 28. **b** ACR50 and ACR70 response rates up to week 28 **c** DAS28(CRP) change from baseline up to week 28. **d** HAQ-DI change from baseline up to week 28 (OL set, imputed data). Footnote: ACR response rates were calculated using NRI if withdrawal was due to an AE or lack or loss of efficacy, and LOCF in case of any other reason. Least squares means (change from baseline) in DAS28(CRP) and HAQ-DI were analyzed using MMRM. *AE* adverse event, *CRP* C-reactive protein, *CZP* certolizumab pegol, *DAS28* Disease Activity Score in 28 joints, *HAQ-DI* Health Assessment Questionnaire-Disability Index, *LOCF* last observation carried forward, *MMRM* mixed-effects model for repeated measures, *NRI* nonresponder imputation, *OL* open-label

The rapid improvements seen in patients receiving CZP therapy during the first 12 weeks of treatment [6] (as measured by ACR20 response rate) were maintained to week 28 (Fig. 2a; Table S1 in Additional file 1). Additionally, in the placebo patients switching to CZP following entry into the OL phase, rapid clinical improvements were seen: by week 20 (8 weeks after OLE entry) placebo → CZP patients showed similar ACR20 response rates to CZP → CZP patients (placebo → CZP: 58.7 %; CZP → CZP: 59.6 %), which were maintained to week 28 (placebo → CZP: 53.3 %; CZP → CZP: 59.7 %). At this time point, placebo → CZP patients and CZP → CZP patients also showed similar ACR50 and ACR70 response rates (Fig. 2b; Table S1 in Additional file 1), improvements in DAS28(CRP) (Fig. 2c; Table S1 in Additional file 1), and improvements in physical function (Fig. 2d; Table S1 in Additional file 1).

### Post hoc analyses by patient subgroups

**Fig. 3:**
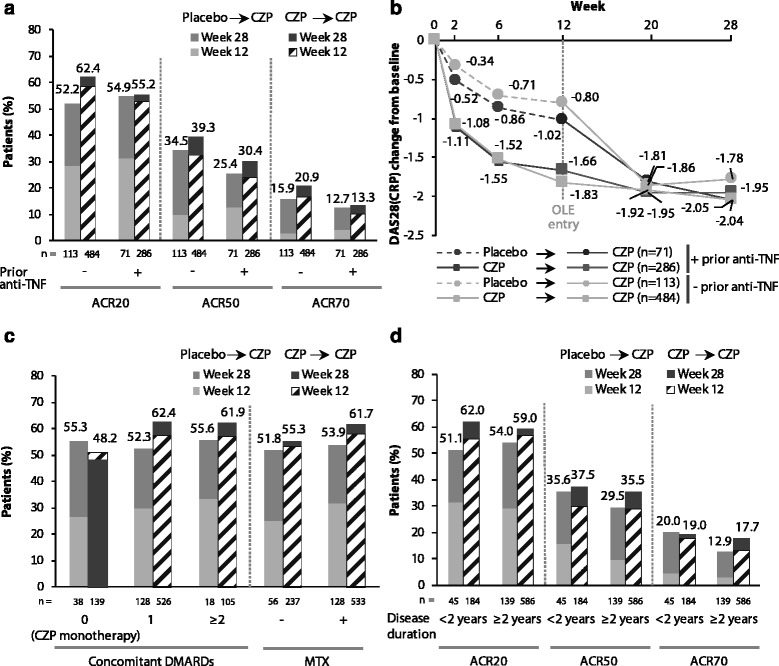
Clinical response at week 28, stratified by baseline characteristics. **a** Week 28 ACR20/ACR50/ACR70 responses stratified by prior anti-TNF therapy. **b** DAS28(CRP) progression up to week 28 by prior anti-TNF therapy. **c** Week 28 ACR20/ACR50/ACR70 responses stratified by baseline DMARDs. **d** Week 28 ACR20/ACR50/ACR70 responses stratified by disease duration (OL set, imputed data). Footnote: ACR response rates were calculated using NRI if withdrawal was due to an AE or lack/loss of efficacy, and LOCF in case of any other reason. Least squares mean (change from baseline) in DAS28(CRP) was analyzed using MMRM. *AE* adverse event, *CRP* C-reactive protein, *CZP* certolizumab pegol, *DAS28* Disease Activity Score in 28 joints, *LOCF* last observation carried forward, *MMRM* mixed-effects model for repeated measures, *NRI* nonresponder imputation, *OL* open-label, *TNF* tumor necrosis factor

At week 28, patients stratified by prior and non-prior anti-TNF use showed similar ACR20, ACR50 and ACR70 response rates in the placebo → CZP and CZP → CZP groups, although there was a numerical trend for slightly higher response rates in the anti-TNF naïve patients (Fig. 3a). ACR20 response rates in patients with vs. without prior anti-TNF were 55.2 % vs. 62.4 % for CZP → CZP patients and 54.9 % vs. 52.2 % for placebo → CZP patients; ACR50 response rates in patients with vs. without prior anti-TNF were 30.4 % vs. 39.3 % for CZP → CZP patients and 25.4 % vs. 34.5 % in placebo → CZP patients). Patients with and without prior anti-TNF use also experienced similar improvements in DAS28(CRP) (Fig. 3b; Table S1 in Additional file 1). ACR20 response rates were also generally similar in the placebo → CZP and CZP → CZP groups when patients were stratified by the number and type of concomitant DMARDs used (0 vs. 1 vs. ≥2 DMARDs; with vs. without MTX use at baseline) (Fig. 3c), although with a trend for numerically higher response rates in patients receiving concomitant DMARD therapy compared to CZP monotherapy (Fig. S1 in Additional file 1). Stratifying patients by their disease duration (<2 vs. ≥2 years) also showed similar response rates. (Fig. 3d). Finally, a trend for improved ACR20 response rates with increasing baseline RF levels (<14 IU/mL (upper limit of normal), 14–50 IU/mL (approximately 3× upper limit of normal [ULN]) and >50 IU/mL) was observed (Fig. S2 in Additional file 1).

### Safety up to week 28

During the DB phase of the study the incidence of AEs and SAEs in the placebo and CZP groups were similar, as reported previously [6], and shown in Table 2. In the OL phase of the study, similar incidences of AEs were reported in placebo → CZP and CZP → CZP patients (77.2 % vs. 67.7 %). The most frequently reported AEs were upper respiratory tract infection, urinary tract infection and flare of RA (Table 3).

**Table 2:** Adverse events: overview of adverse events in the DB and OL phases

	DB phase week 0 – week 12		OL phase week 12 – week 28	
	(Safety set)		(OL set)	
	Placebo	CZP	Placebo → CZP^a^	CZP → CZP^a^
	(n = 209)	(n = 846)	(n = 184)	(n = 770)
Any AE, incidence/100 pt-yrs (n, %)	483.2 (129, 61.7)	522.1 (571, 67.5)	328.9 (142, 77.2)	239.1 (521, 67.7)
Patients discontinuing due to AE, n (%)	8 (3.8)	40 (4.7)	3 (1.6)	26 (3.4)
Injection and infusion site reactions, incidence/100 pt-yrs (n, %)	4.2 (2, 1.0)	25.3 (49, 5.8)	8.8 (9, 4.9)	3.2 (14, 1.8)
Serious AEs, incidence/100 pt-yrs (n, %)	25.8 (12, 5.7)	26.7 (52, 6.1)	20.6 (21, 11.4)	13.0 (56, 7.3)
Serious infections, incidence/100 pt-yrs (n, %)	8.3 (4, 1.9)	11.1 (22, 2.6)	5.7 (6, 3.3)	4.1 (18, 2.3)
Deaths, n (%)	0	2 (0.2)	1 (0.5)	1 (0.1)

*DB* double-blind, *OL* open-label, *CZP* certolizumab pegol, *AE* adverse event, *Pt-yrs* patient-years, *n* number of patients reporting AE

^a^Patients who completed 12 weeks of treatment with either CZP 200 mg every other week (Q2W) or placebo during the double-blind phase entered the OL phase and subsequently received active treatment (CZP 200 mg Q2W) for ≥16 weeks

**Table 3:** Adverse events: most common adverse events reported in the OL phase

MedDRA v 9.0 System organ class High level term	OL phase week 12 – week 28	
	(OL set)	
	Placebo → CZP^a^	CZP → CZP^a^
	(n = 184)	(n = 770)
Most common AEs, incidence/100 pt-yrs (n, %)^b^:		
Infections and infestations	92.9 (73, 39.7)	79.4 (271, 35.2)
Nasopharyngitis	12.9 (13, 7.1)	9.3 (40, 5.2)
Sinusitis	10.8 (11, 6.0)	7.9 (34, 4.4)
Upper respiratory tract infection	16.0 (16, 8.7)	14.2 (60, 7.8)
Urinary tract infection	14.8 (15, 8.2)	11.9 (51, 6.6)
Musculoskeletal and connective tissue disorders	47.4 (178, 23.1)	49.1 (227, 23.8)
Rheumatoid arthritis	18.0 (18, 9.8)	12.4 (53, 6.9)
Nervous system disorders	26.0 (25, 13.6)	15.6 (66, 8.6)
Headache	10.8 (11, 6.0)	5.5 (24, 3.1)
Skin and subcutaneous tissue disorders	33.3 (31, 16.8)	22.5 (93, 12.1)
Rash	10.9 (11, 6.0)	5.5 (24, 3.1)

*OL* open-label, *CZP* certolizumab pegol, *AE* adverse event, *Pt-yrs* patient-years, *n* number of patients reporting AE

^a^Patients who completed 12 weeks of treatment with either CZP 200 mg every other week (Q2W) or placebo during the double-blind phase entered the OL phase and subsequently received active treatment (CZP 200 mg Q2W) for ≥16 weeks

^b^AEs occurring in ≥5 % of patients in either treatment group are presented

During the OL phase, four of the placebo → CZP patients (2.2 %) and 29 (3.8 %) of the CZP → CZP patients withdrew from the study due to AEs. The most common AEs leading to withdrawal were Herpes zoster infections (two patients overall; 0.2 %), rheumatoid arthritis (two patients; 0.2 %) and respiratory tract small cell carcinomas (two patients; 0.2 %). Injection and infusion site reactions occurred in 4.9 % of CZP → CZP patients and 1.8 % of placebo → CZP patients; there were no incidences of serious infusion site reactions.

SAEs were experienced by 11.4 % of patients in the placebo → CZP group and 7.3 % of patients in the CZP → CZP group during the OL phase, the most common of which were lower respiratory tract and lung infections, reported by four patients (2.2 %) and seven patients (0.9 %) in the placebo → CZP and CZP → CZP groups, respectively. There were no reported cases of tuberculosis or other opportunistic infections. There was one death in the CZP → CZP group (due to small cell lung cancer) and one death in the placebo → CZP group (myocardial infarction).

### Predictability analyses

#### *Predictability of achieving LDA by cumulative nonresponse in DAS28*(*ESR*)*, CDAI, and SJC up to week 12*

At week 28, 225 patients (27.4 %) from the FAS achieved DAS28(ESR) LDA. Failure to achieve LDA was associated with the timing of nonresponse (as measured by DAS28(ESR), CDAI and percentage change in SJC) up to week 12 (Table 4). Reductions in DAS28(ESR) of <1.2 up to weeks 2, 6 and 12 were associated with a chance of LDA at week 28 of 24.8 %, 16.8 % and 5.4 %, respectively. Similarly, SJC percentage changes of <25 % up to weeks 2, 6 and 12 were associated with a chance of LDA at week 28 of 20.7 %, 11.9 % and 5.9 %, respectively (at week 12, LS mean change from baseline [standard error] SJC was −6.3 [0.20] in CZP patients, −3.9 [0.36] in placebo patients). The same trend was seen based on early CDAI nonresponse: patients with CDAI reductions <10 by these time points had 25.3 %, 18.8 % and 7.1 % chance of LDA at week 28 (Table 4). Patients who did achieve a reduction from baseline in DAS28(ESR) of ≥1.2, SJC ≥25 % or CDAI ≥10 at any week up to week 12 had a higher chance of achieving week 28 LDA (35.6 %, 36.1 % and 34.1 %, respectively).

**Table 4:**
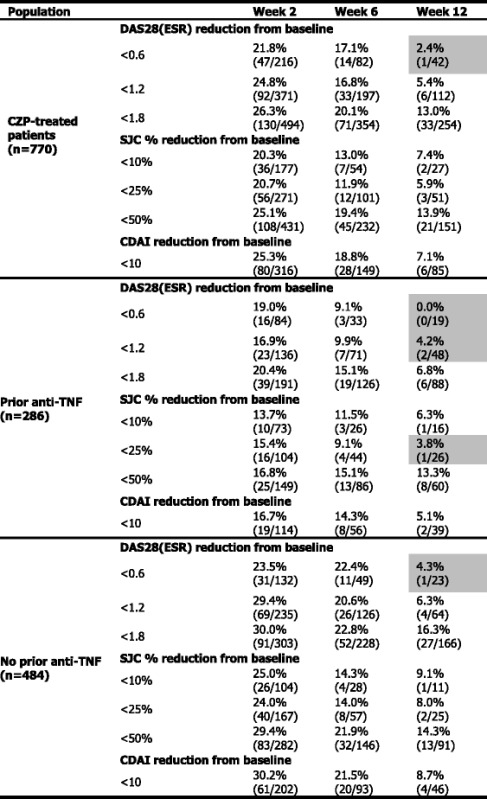
Proportion of CZP-treated patients achieving LDA at week 28. Predictability by change in DAS28(ESR), SJC and CDAI up to the indicated week – overall population and by prior anti-TNF experience (OL set, observed data)

Shading represents ≤5.0 % of patients achieving LDA (DAS28[ESR] ≤3.2) at week 28

Numbers in brackets are the number of patients who achieved LDA at week 28, over the number of patients not achieving the SJC %, DAS28(ESR) or CDAI change threshold at any week up to the week presented

*CZP* certolizumab pegol, *OL* open-label, LDA low disease activity, *DAS28* Disease Activity Score in 28 joints, *CRP* C-reactive protein, *SJC* swollen joint count, *CDAI* Clinical Disease Activity Index, *TNF* tumor necrosis factor, *ESR* erythrocyte sedimentation rate

Failure to achieve LDA at week 28 was also associated with the magnitude of early response. Patients who failed to meet the lower thresholds for improvement had a lower chance of achieving LDA at week 28: reductions from baseline in DAS28(ESR) of <0.6, <1.2 and <1.8 up to week 12 were predictive of a 2.4 %, 5.4 % and 13.0 % chance of achieving LDA at week 28, respectively (Table S2 in Additional file 1). Correspondingly, patients achieving <10 %, <25 % and <50 % reductions from baseline in SJC had 7.4 %, 5.9 % and 13.9 % chance of week 28 LDA. For any given threshold, the failure to respond by a later time point was associated with a lower chance of achieving LDA at week 28. Similar trends were seen when LOCF imputation was used to analyze data from the FAS (Table S2 in Additional file 1).

Results were confirmed in sensitivity analyses, in which DAS28(ESR) nonresponse was analyzed ‘at’, rather than ‘up to’, each time point. The same trend was observed: in CZP-treated patients, the percentage achieving LDA at week 28 for any given threshold in DAS28(ESR) was lower in nonresponders at later time points (Table S3 in Additional file 1).

#### Predictability of achieving LDA stratified by prior anti-TNF experience

**Fig. 4:**
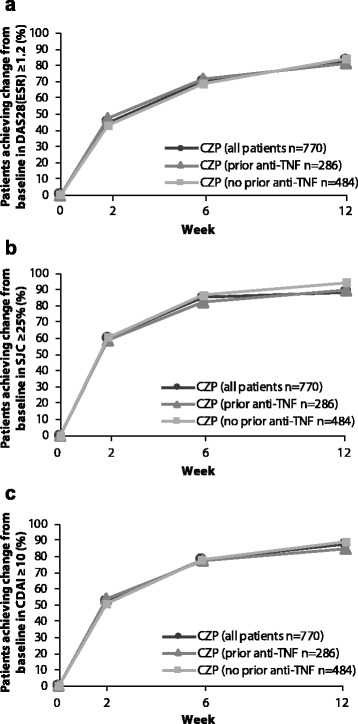
Clinical improvements in CZP-treated patients to week 12, stratified by prior-anti-TNF exposure. Cumulative proportion of CZP patients achieving (**a**) ≥1.2 reduction from baseline in DAS28(ESR), (**b**) ≥25 % change in baseline in SJC and (**c**) ≥10 reduction from baseline in CDAI (FAS, LOCF imputation). Footnote: Patients could achieve the response at any time point up to and including the relevant week. If all prior weeks were missing the patient was not counted. *CDAI* Clinical Disease Activity Index, *CZP* certolizumab pegol, *DAS28* Disease Activity Score in 28 joints, *ESR* erythrocyte sedimentation rate, *FAS* full analysis set, *LOCF* last observation carried forward, *SJC* swollen joint count, *TNF* tumor necrosis factor

The proportion of patients achieving a change from baseline in DAS28(ESR) ≥1.2, SJC ≥25 % or CDAI ≥10 by week 12 was similar between patients who had received prior anti-TNF therapy and those who had not (Fig. 4). For any given threshold in DAS28(ESR), SJC or CDAI, failure to respond up to a later time point was associated with a lower chance of achieving at week 28, regardless of prior anti-TNF experience (Table 4).

## Discussion

In a diverse group of patients with active RA, addition of CZP to current therapy for up to 28 weeks was associated with sustained improvements in disease activity and physical function as measured by a variety of clinical endpoints, including ACR20/ACR50/ACR70 response rates, DAS28(ESR), DAS28(CRP), and HAQ-DI.

By week 20, patients who began receiving CZP at week 12 (following placebo treatment in the DB phase) showed similar improvements in disease activity to those who had received CZP since week 0. Both the onset and the magnitude of response were consistent with previous studies, in which clinical benefits associated with CZP treatment have been observed from week 1 following the commencement of CZP therapy, both as a monotherapy (the FAST4WARD study) [10], and in addition to MTX (the RAPID 1 and 2 studies) [11, 12]. Sustained efficacy was observed across all subgroups of CZP patients, including those with and without prior anti-TNF use, receiving CZP monotherapy or concomitant DMARDs (regardless of type or number), with and without concomitant MTX use at baseline, and with disease duration of < or ≥2 years. This is in agreement with the DOSEFLEX study of CZP, where CZP exhibited similar efficacy in RA patients with or without prior exposure to anti-TNF agents over 34 weeks [13]. Previously published studies employing a range of biologic agents have also demonstrated the efficacy of this class of drug in RA patients who have previously shown inadequate responses to anti-TNF agents. These include both TNF-targeting (golimumab) and non-TNF-targeting (rituximab, tocilizumab, abatacept) therapies [14–17]. Moreover, in the current study, there was a suggestion of a possible correlation between improved ACR20 response and increasing baseline RF levels, based upon the observed data. Previous reports suggested that RF negativity may be associated with greater response to monoclonal anti-TNFs [18–20], but these findings have been inconsistent, with other reports suggesting no strong association [21]. This suggests that individual factors may be insufficient to predict clinical response, and in future the identification and validation of multifactorial “smart biomarkers” may facilitate recognition of those patients who are likely to have the desired clinical response, allowing the most appropriate treatment options to be selected [22].

The safety profile observed here was similar to previous CZP trials [10–12], but with no tuberculosis or opportunistic infections recorded. The safety profile was in line with the anti-TNF class [23, 24]. No new safety signals were identified. The incidences of AEs and SAEs were similar between the CZP and placebo groups during the 12-week DB phase [6], and the CZP → CZP and placebo → CZP groups during the OL phase.

In this diverse patient population, the majority of patients responded to treatment with CZP by week 12. Low chances of achieving LDA at week 28 could be predicted for subgroups early in the course of CZP treatment based on the timing and magnitude of the initial change in DAS28(ESR). These data are in line with the post hoc analysis of the RAPID 1 study of CZP in adult patients with active RA despite MTX therapy, who received CZP 200 mg or 400 mg Q2W plus MTX [7]. These analyses demonstrated that failure to achieve improvement in DAS28 within the first 12 weeks of CZP therapy was predictive of a low probability of achieving LDA at year 1. Moreover, the percentage of patients achieving LDA at week 28 in subgroups of nonresponders at early time points was found to be strongly dependent on the magnitude and timing of the lack of the response. This is in agreement with published studies of other biologics, which report that failure to respond by later time points (up to 26 weeks after commencing biologic therapy) correlated with reduced likelihood of achieving clinical endpoints. In the present study, a low percentage of patients achieving LDA at week 28 could also be predicted using either SJC or CDAI early nonresponses. Furthermore, a lack of response up to week 12, as measured either by change from baseline in DAS28(ESR) or CDAI, or percentage change from baseline in SJC, predicted failure to achieve LDA at week 28 in both patients naïve to, and previously exposed to, anti-TNFs.

These data support the concept that a decision can be made at week 12 regarding continuation of CZP treatment. This is in line with the treat-to-target recommendations, which state that until the desired treatment target is reached, drug therapy should be adjusted at least every 3 months [25].

Limitations of this study included the lack of radiographic assessment, without which any joints swollen due to factors other than structural damage could not be detected. Furthermore, results are reported here up to week 28 and longer-term data would be useful in ascertaining the maintenance of response. Predictability analyses were conducted using the OL set, comprising all patients who received at least one dose of CZP in the OL phase, and also using the FAS, comprising all patients who were administered at least one dose of CZP, and which included 80 patients (9.4 %) who dropped out of the study by week 12. While the predictive values appeared similar when calculated either using LOCF imputation or an observed case analysis, both ways may slightly bias the predicted response rates, and as such, general trends may be of more clinical value than precise figures.

## Conclusions

In conclusion, the results of the REALISTIC study conducted in patients reflective of real-world clinical care demonstrate sustained improvements in disease activity and physical functions following 28 weeks of CZP treatment across this diverse population of RA patients, including with and without prior anti-TNF use, on a variety of concomitant DMARDs and regardless of disease duration (< and ≥2 years). Additionally, in patients both naïve to and previously exposed to prior anti-TNFs, DAS28(ESR), SJC or CDAI non-response at time points up to week 12 were predictive of a low probability of achieving LDA at week 28.

### Consent statement

The study protocol was approved by the Institutional Review Board/Independent Ethics Committee as defined in local regulations and performed according to the Declaration of Helsinki. All patients provided written consent.

### Ethics statement

The following ethics committees approved this study: Capital Health, Halifax, NS, Canada; CEIC Hospital Clinic I Provincial, Barcelona, Spain; CHU Pontchaillou, Rennes, France; College of Physicians and Surgeons of Alberta, Edmonton, AL, Canada; Comitato Etico locale per Sperimentazione Clinica dei Medicinali Azienda Ospedaliera Universitaria Senese, Siena, Italy; Commissie Medische Ethiek, Leiden, the Netherlands; Conjoint Health Research Ethics Board, Calgary, AL, Canada; Ethikkommission der Ärztekammer Hamburg, Hamburg, Germany; Hôpital Maisonneuve Rosemont, Montreal, QC, Canada; Institutional Board of Research Associates New York, New York, NY, USA; IUPUI Clarian Institutional Review Board, Indianapolis, IN, USA; Loyola University Medical Center, Maywood, IL, USA; Mayo Foundation IRB, Rochester, MN, USA; McGuire Institutional Review Board, Richmond, VA, USA; Mount Sinai Hospital, Toronto, ON, Canada; Office of Human Subjects Research IRB, Baltimore, MD, USA; Oklahoma Medical Research Foundation; Oklahoma City, OK, USA; Partners Human Research Committee, Boston, MA, USA; Quorum Review, Inc., Seattle, WA, USA; Robert Wood Johnson Medical School, New Brunswick, NJ, USA; St Joseph’s Mercy Health Center, Hot Springs, AR, USA; St Luke’s IRB, Duluth, MN, USA; Sutter Health Central Area, Sacramento, CA, USA; Texas Health Resources IRB, Arlington, TX, USA; The University of Arizona, Tucson, AZ, USA; The University of North Carolina, Chapel Hill, NC, USA; The University of Texas, Houston, TX, USA; The University of Western Ontario, London, ON, Canada; Thomas Jefferson University IRB, Philadelphia, PA, USA; UCSD, La Jolla, CA, USA; University Health Network, Toronto, ON, Canada; University of British Columbia, Vancouver, BC, Canada; University of Illinois College, Peoria, IL, USA; University of Manitoba Bannatyne Campus, Winnipeg, MB, Canada; University of North Texas Health, Fort Worth, TX, USA; University of Utah, Salt Lake City, UT, USA; Western Institutional Review Board, Olympia, WA, USA.

## Additional file

Additional file 1:
**Additional figures and tables (two figures, three tables).** (DOCX 112 kb)

## Abbreviations

ACR: American College of Rheumatology
AE: Adverse event
CDAI: Clinical Disease Activity Index
CRP: C-reactive protein
CZP: Certolizumab pegol
DAS: Disease activity score
DB: Double-blind
DMARD: Disease-modifying antirheumatic drug
DOSEFLEX: Dosing flexibility
ESR: Erythrocyte sedimentation rate
FAS: Full analysis set
FAST4WARD: eFficAcy and Safety of cerTolizumab pegol – 4 weekly dosAge in RheumatoiD arthritis
HAQ-DI: Health Assessment Questionnaire-Disability Index
LDA: Low disease activity
LOCF: Last observation carried forward
MTX: Methotrexate
MMRM: Mixed-effects model for repeated measures
NRI: Nonresponder imputation
NSAID: Nonsteroidal anti-inflammatory drug
OL: Open-label
Q2W: Every other week
RA: Rheumatoid arthritis
RAPID 1: Rheumatoid Arthritis Prevention of Structural Damage
REALISTIC: RA Evaluation in Subjects Receiving TNF Inhibitor CZP
RF: Rheumatoid factor
SAE: Serious adverse event
SDAI: Simplified Disease Activity Index
SJC: Swollen joint count
TB: Tuberculosis
TJC: Tender joint count
TNF: Tumor necrosis factor
ULN: Upper limit of normal
