# Supplementary material for: Twenty-eight-week results from the REALISTIC phase IIIb randomized trial: efficacy, safety and predictability of response to certolizumab pegol in a diverse rheumatoid arthritis population
Source: Arthritis Res Ther. 2015 Nov 15;17:325. doi: 10.1186/s13075-015-0841-9 (PMC4644627; doi:10.1186/s13075-015-0841-9)

**ADDITIONAL MATERIAL**

**Additional Table 1.** Clinical outcomes at Week 28 (OL set, imputed data)

| **Change from baseline** | **OL set** | |
| --- | --- | --- |
|  | Placebo🡪CZP^a^ | CZP 🡪 CZP^a^ |
| ACR20 response rate |  |  |
| All patients | 53.3% (n=184) | 59.7% (n=770) |
| + prior anti-TNF | 54.9% (n=71) | 55.2% (n=286) |
| - prior anti-TNF | 52.2% (n=113) | 62.4% (n=484) |
| ACR50 response rate |  |  |
| All patients | 31.0% (n=184) | 36.0% (n=770) |
| + prior anti-TNF | 25.4% (n=71) | 30.4% (n=286) |
| - prior anti-TNF | 34.5% (n=113) | 39.3% (n=484) |
| ACR70 response rate |  |  |
| All patients | 14.7% (n=184) | 18.1% (n=770) |
| + prior anti-TNF | 12.7% (n=71) | 13.3% (n=286) |
| - prior anti-TNF | 15.9% (n=113) | 20.9% (n=484) |
| DAS28(CRP), LS mean (SE) |  |  |
| All patients | -1.82 (0.103)  (n=184) | -1.97 (0.059)  (n=770) |
| + prior anti-TNF | -2.04 (0.171)  (n=71) | -1.95 (0.107)  (n=286) |
| - prior anti-TNF | -1.78 (0.127)  (n=113) | -2.05 (0.065)  (n=484) |
| CDAI, LS mean (SE) | -20.75 (1.006)  (n=184) | -21.88 (0.572)  (n=770) |
| HAQ-DI, LS mean (SE) | -0.40 (0.042)  (n=184) | -0.47 (0.024)  (n=770) |
| ^a^Patients who completed 12 weeks of treatment with either CZP 200 mg Q2W or placebo during the double-blind phase entered the OLE phase and subsequently received active treatment (CZP 200 mg Q2W) for ≥16 weeks DAS28(CRP), CDAI and HAQ-DI LS means were calculated using MMRM imputation; ACR20, ACR50 and ACR70 response rates were calculated using NRI if withdrawal was due to an AE or lack/loss of efficacy, and LOCF in case of any other reason  LS mean: least squares mean; SE: standard error | | |

**Additional Table 2.** Proportion of CZP-treated patients achieving LDA at Week 28 by DAS28(ESR) change up to the indicated Week – Overall population and by prior anti-TNF experience (FAS, LOCF imputation)

| Population | | Week 2 | Week 6 | Week 12 |
| --- | --- | --- | --- | --- |
| CZP-treated patients (n=851) | DAS28(ESR) reduction from baseline | | | |
|  | <0.6 | 17.9%  (48/268) | 12.2%  (14/115) | 1.4%  (1/70) |
|  | <1.2 | 20.9%  (92/440) | 13.8%  (35/254) | 3.9%  (6/154) |
|  | <1.8 | 22.4%  (132/588) | 17.1%  (75/439) | 10.5%  (34/323) |
| Prior anti-TNF  (n=320) | DAS28(ESR) reduction from baseline | | | |
|  | <0.6 | 14.0%  (14/100) | 4.7%  (2/43) | 0%  (0/30) |
|  | <1.2 | 13.0%  (21/161) | 7.8%  (7/90) | 3.2%  (2/63) |
|  | <1.8 | 16.5%  (38/230) | 12.5%  (20/160) | 6.0%  (7/117) |
| No prior anti-TNF  (n=531) | DAS28(ESR) reduction from baseline | | | |
|  | <0.6 | 20.2%  (34/168) | 16.7%  (12/72) | 2.5%  (1/40) |
|  | <1.2 | 25.4%  (71/279) | 17.1%  (28/164) | 4.4%  (4/91) |
|  | <1.8 | 26.3%  (94/358) | 19.7%  (55/279) | 13.1%  (27/206) |
| Shading represents ≤5.0% of patients achieving LDA (DAS28[ESR] ≤3.2) at Week 28  Numbers in brackets are the number of patients who achieved LDA at Week 28 over the number of patients not achieving the DAS28(ESR) change threshold up to the week presented | | | | |

**Additional Table 3.** Proportion of CZP-treated patients achieving LDA at Week 28 by DAS28(ESR) change at the indicated Week – Overall population and by prior anti-TNF experience (FAS, LOCF imputation)

| Population | | Week 2 | Week 6 | Week 12 |
| --- | --- | --- | --- | --- |
| CZP-treated patients (n=851) | DAS28(ESR) reduction from baseline | | | |
|  | <0.6 | 17.9% (48/268) | 13.3% (21/158) | 6.2% (9/146) |
|  | <1.2 | 20.9% (92/440) | 14.4% (44/305) | 7.3% (19/260) |
|  | <1.8 | 22.4% (132/558) | 17.8% (85/478) | 11.9% (49/411) |
| Prior anti-TNF  (n=320) | DAS28(ESR) reduction from baseline | | | |
|  | <0.6 | 14.0% (14/100) | 6.3%  (4/63) | 8.5% (6/71) |
|  | <1.2 | 13.0% (21/161) | 9.7% (11/113) | 7.1% (8/112) |
|  | <1.8 | 16.5% (38/230) | 14.1% (25/177) | 9.7% (16/165) |
| No prior anti-TNF  (n=531) | DAS28(ESR) reduction from baseline | | | |
|  | <0.6 | 20.2% (34/168) | 17.9% (17/95) | 4.0% (3/75) |
|  | <1.2 | 25.4% (71/279) | 17.2% (33/192) | 7.4% (11/148) |
|  | <1.8 | 26.3% (94/358) | 19.9% (60/301) | 13.4% (33/246) |
| Shading represents ≤5.0% of patients achieving LDA (DAS28[ESR] ≤3.2) at Week 28  Numbers in brackets are the number of patients who achieved LDA at Week 28 over the number of patients not achieving the DAS28(ESR) change threshold at the week presented | | | | |

**Additional Figure 1.** Week 28 ACR20/ACR50/ACR70 responses by concomitant DMARD use at baseline (OL set, imputed data)

**
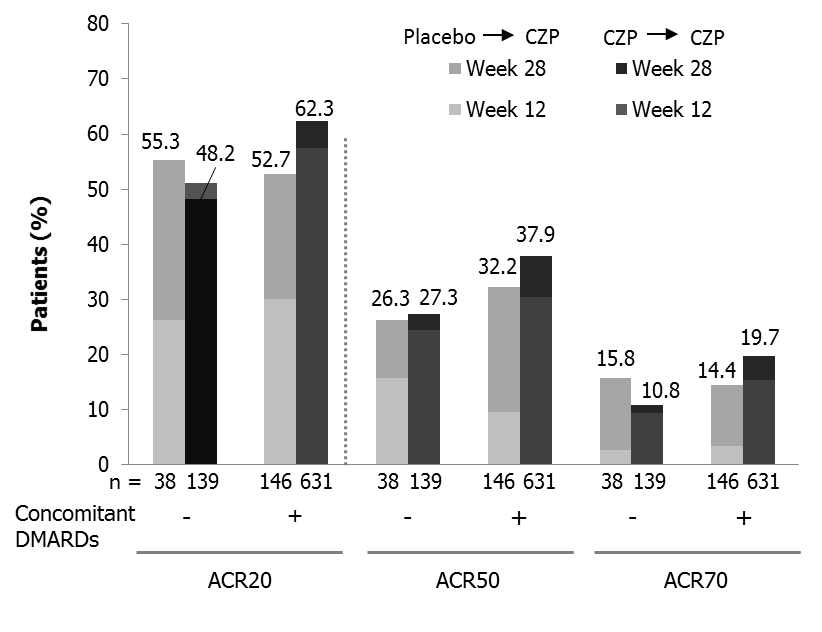
**

All analyses were conducted on the OL set. ACR response rates were calculated using NRI if withdrawal was due to an AE or lack/loss of efficacy, and LOCF in case of any other reason

**Additional Figure 2.** Week 28 ACR20, ACR50 and ACR70 responses by RF status at baseline (OL set, NRI imputation)


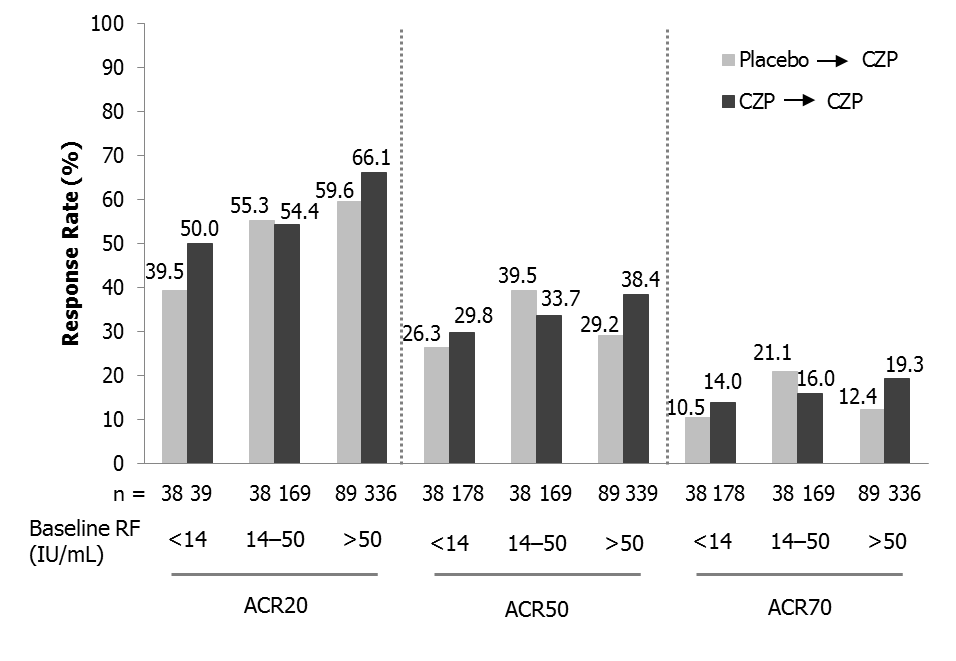

Supplement: Additional file 1: — Additional figures and tables (two figures, three tables). (DOCX 112 kb) [file 13075_2015_841_MOESM1_ESM.docx]
